# 1-Butyl­pyridinium bis­(1,2-dicyano­ethene-1,2-dithiol­ato)nickelate(III)

**DOI:** 10.1107/S1600536811042103

**Published:** 2011-10-22

**Authors:** Hai-Bao Duan

**Affiliations:** a School of Biochemical and Environmental Engineering, Nanjing Xiaozhuang College, Nanjing 210017, People’s Republic of China

## Abstract

The Ni^III^ atom in the anion of the title complex, (C_9_H_14_N)[Ni(C_4_N_2_S_2_)_2_], is coordinated by four S atoms of two maleonitrile­dithiol­ate ligands, and exhibits a square-planar coordination geometry.

## Related literature

For background to designed functional materials, see: Nishijo *et al.* (2000 ▶); Robertson & Cronin (2002 ▶); Ni *et al.* (2005 ▶). For related structures, see: Ni *et al.* (2004 ▶); Ren *et al.* (2004 ▶, 2008 ▶); Duan *et al.* (2010 ▶). For the synthesis of disodium maleonitrile­dithiol­ate and 1-butane-pyridinium bromide, see: Davison & Holm (1967 ▶); Yao *et al.* (2008 ▶).
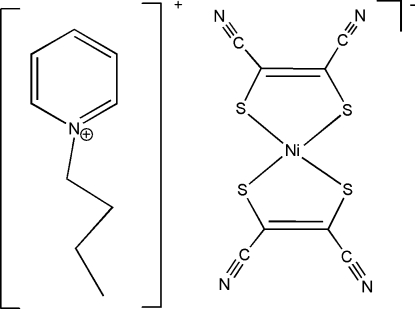


## Experimental

### 

#### Crystal data

(C_9_H_14_N)[Ni(C_4_N_2_S_2_)_2_]
*M* *_r_* = 475.30
Triclinic, 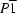

*a* = 9.2764 (11) Å
*b* = 9.9863 (11) Å
*c* = 12.7115 (15) Å
α = 81.695 (9)°
β = 75.882 (10)°
γ = 64.480 (11)°
*V* = 1029.5 (2) Å^3^
*Z* = 2
Cu *K*α radiation
μ = 5.25 mm^−1^
*T* = 293 K
0.3 × 0.1 × 0.1 mm

#### Data collection

Bruker SMART CCD area-detector diffractometer
Absorption correction: multi-scan (*SADABS*; Sheldrick, 2002) ▶ *T*_min_ = 0.559, *T*_max_ = 0.591
7461 measured reflections
3196 independent reflections
2499 reflections with *I* > 2σ(*I*)
*R*_int_ = 0.018

#### Refinement

*R*[*F* ^2^ > 2σ(*F* ^2^)] = 0.036
*wR*(*F* ^2^) = 0.109
*S* = 1.04
3196 reflections
245 parameters
1 restraint
H-atom parameters constrained
Δρ_max_ = 0.36 e Å^−3^
Δρ_min_ = −0.33 e Å^−3^

### 

Data collection: *SMART* (Bruker, 2000 ▶); cell refinement: *SAINT* (Bruker, 2000 ▶); data reduction: *SAINT*; program(s) used to solve structure: *SHELXTL* (Sheldrick, 2008 ▶); program(s) used to refine structure: *SHELXTL*; molecular graphics: *SHELXTL*; software used to prepare material for publication: *SHELXTL*.

## Supplementary Material

Crystal structure: contains datablock(s) global, I. DOI: 10.1107/S1600536811042103/tk2798sup1.cif

Structure factors: contains datablock(s) I. DOI: 10.1107/S1600536811042103/tk2798Isup2.hkl

Additional supplementary materials:  crystallographic information; 3D view; checkCIF report

## Figures and Tables

**Table 1:** Selected bond lengths (Å)

Ni1—S1	2.1501 (8)
Ni1—S2	2.1436 (8)
Ni1—S3	2.1458 (8)
Ni1—S4	2.1461 (8)

**Fig. 1.**
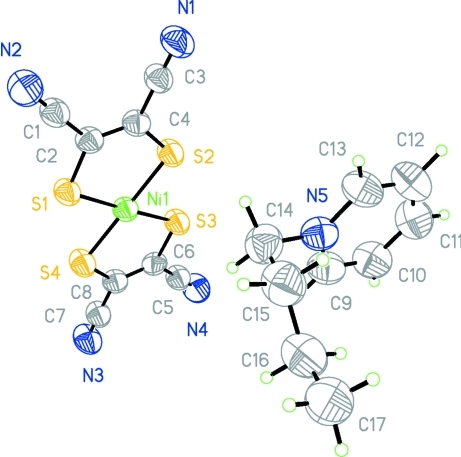
The molecular structures of the ionic components of (I), showing the atom-numbering scheme and displacement ellipsoids at the 30% probability level.

## supplementary crystallographic information

### Comment

Supramolecular chemistry and molecular crystal engineering, which is the planning and utilization of crystal-oriented syntheses for the bottom-up construction of functional molecular solids from molecules and ions, are powerful tools for the assembly of designed functional materials (Robertson & Cronin, 2002). Bis-1,2-dithiolene complexes of transition metals have been widely studied due to their novel properties and applications in the areas of near-infrared (near-IR) dyes, conducting, magnetic and non-linear optical materials (Nishijo *et al.*, 2000; Ni *et al.*, 2005). These applications arise due to a combination of functional properties, specific geometries and intermolecular interactions. Herein, we report the crystal structure of the title compound (I).

The molecular structure of (I) is illustrated in Fig. 1. and selected bond distances are given in Table 1. The asymmmetric units comprises one [Ni(mnt)_2_]^-^ monoanion and one 1-butyl-pyridinium cation. The Ni ion in the [Ni(mnt)_2_]^-^ anion is coordinated by four sulfur atoms of two mnt^2-^ ligands, and exhibits square-planar coordination geometry. The bond lengths in the anion are in good agreement with those found in other [Ni(mnt)_2_]^-^ compounds (Ni *et al.*, 2004; Ren *et al.*, 2004; Duan *et al.*, 2010; Ren *et al.*, 2008).

### Experimental

All reagents and chemicals were purchased from commerical sources and used without further purification. The starting materials disodium maleonitriledithiolate (Davison *et al.*, 1967) and 1-butyl-pyridinium bromide (Yao *et al.*, 2008) were synthesized following the literature procedures. Disodium maleonitriledithiolate (456 mg, 2.5 mmol) and nickel chloride hexahydrate (297 mg, 1.25 mmol) were mixed under stirring in water (20 ml) at room temperature. Subsequently, a solution of 1-butyl-pyridinium bromide (1.5 mmol) in methanol (10 ml) was added to the mixture. The red precipitate that was immediately formed was filtered off and washed with methanol. Then, a methanol solution of I_2_ (205 mg, 0.8 mmol) was added slowly. After stirring for 40 minutes, the mixture was allowed to stand overnight. The microcrystals formed were recrystallized from acetone to give black blocks.

### Refinement

The C-bound H atoms were geometrically placed (C—H = 0.93–0.97 Å) and refined as riding with *U_iso_*(H) = 1.2–1.5*U_eq_*(C).

### Crystal data

**Table:**

(C_9_H_14_N)[Ni(C_4_N_2_S_2_)_2_]	*V* = 1029.5 (2) Å^3^
*M**_r_* = 475.30	*Z* = 2
Triclinic, *P*1	*F*(000) = 486
Hall symbol: -P 1	*D*_x_ = 1.533 Mg m^−^^3^
*a* = 9.2764 (11) Å	Cu *K*α radiation, λ = 1.54178 Å
*b* = 9.9863 (11) Å	µ = 5.25 mm^−^^1^
*c* = 12.7115 (15) Å	*T* = 293 K
α = 81.695 (9)°	Block, black
β = 75.882 (10)°	0.3 × 0.1 × 0.1 mm
γ = 64.480 (11)°	

### Data collection

**Table:**

Bruker SMART CCD area-detector diffractometer	3196 independent reflections
Radiation source: fine-focus sealed tube	2499 reflections with *I* > 2σ(*I*)
graphite	*R*_int_ = 0.018
φ and ω scans	θ_max_ = 62.6°, θ_min_ = 3.6°
Absorption correction: multi-scan (*SADABS*; Sheldrick, 2002)	*h* = −10→9
*T*_min_ = 0.559, *T*_max_ = 0.591	*k* = −11→11
7461 measured reflections	*l* = −14→12

### Refinement

**Table:**

Refinement on *F*^2^	Primary atom site location: structure-invariant direct methods
Least-squares matrix: full	Secondary atom site location: difference Fourier map
*R*[*F*^2^ > 2σ(*F*^2^)] = 0.036	Hydrogen site location: inferred from neighbouring sites
*wR*(*F*^2^) = 0.109	H-atom parameters constrained
*S* = 1.04	*w* = 1/[σ^2^(*F*_o_^2^) + (0.0752*P*)^2^] where *P* = (*F*_o_^2^ + 2*F*_c_^2^)/3
3196 reflections	(Δ/σ)_max_ < 0.001
245 parameters	Δρ_max_ = 0.36 e Å^−^^3^
1 restraint	Δρ_min_ = −0.33 e Å^−^^3^

### Special details

**Table:**

Geometry. All e.s.d.'s (except the e.s.d. in the dihedral angle between two l.s. planes) are estimated using the full covariance matrix. The cell e.s.d.'s are taken into account individually in the estimation of e.s.d.'s in distances, angles and torsion angles; correlations between e.s.d.'s in cell parameters are only used when they are defined by crystal symmetry. An approximate (isotropic) treatment of cell e.s.d.'s is used for estimating e.s.d.'s involving l.s. planes.
Refinement. Refinement of *F*^2^ against ALL reflections. The weighted *R*-factor *wR* and goodness of fit *S* are based on *F*^2^, conventional *R*-factors *R* are based on *F*, with *F* set to zero for negative *F*^2^. The threshold expression of *F*^2^ > σ(*F*^2^) is used only for calculating *R*-factors(gt) *etc*. and is not relevant to the choice of reflections for refinement. *R*-factors based on *F*^2^ are statistically about twice as large as those based on *F*, and *R*- factors based on ALL data will be even larger.

### Fractional atomic coordinates and isotropic or equivalent isotropic displacement parameters (Å^2^)

**Table:**

	*x*	*y*	*z*	*U*_iso_*/*U*_eq_	
Ni1	0.17351 (5)	0.21297 (4)	0.32522 (3)	0.05459 (18)	
S1	−0.01043 (8)	0.19088 (7)	0.45807 (6)	0.0601 (2)	
S2	0.04693 (8)	0.45024 (7)	0.31675 (6)	0.0634 (2)	
S3	0.34944 (8)	0.23715 (8)	0.18766 (6)	0.0646 (2)	
S4	0.30030 (9)	−0.02462 (7)	0.33157 (6)	0.0681 (2)	
N1	−0.3268 (3)	0.7577 (3)	0.4479 (2)	0.0791 (7)	
N2	−0.4145 (3)	0.4193 (3)	0.6218 (2)	0.0862 (8)	
N3	0.6747 (3)	−0.3235 (3)	0.1797 (2)	0.0886 (8)	
N4	0.7294 (3)	0.0134 (3)	0.0023 (2)	0.0882 (8)	
N5	0.1415 (3)	0.7746 (2)	0.81191 (17)	0.0575 (5)	
C1	−0.2966 (3)	0.3967 (3)	0.5591 (2)	0.0611 (7)	
C2	−0.1507 (3)	0.3709 (3)	0.4772 (2)	0.0531 (6)	
C3	−0.2387 (3)	0.6364 (3)	0.4331 (2)	0.0599 (7)	
C4	−0.1257 (3)	0.4844 (3)	0.4169 (2)	0.0550 (6)	
C5	0.6208 (3)	0.0325 (3)	0.0733 (2)	0.0652 (7)	
C6	0.4856 (3)	0.0568 (3)	0.1630 (2)	0.0565 (6)	
C7	0.4645 (3)	−0.0567 (3)	0.2251 (2)	0.0569 (6)	
C8	0.5794 (3)	−0.2072 (3)	0.2023 (2)	0.0659 (7)	
C9	0.2814 (3)	0.6993 (3)	0.7434 (2)	0.0650 (7)	
H9	0.3298	0.5961	0.7497	0.078*	
C10	0.3530 (4)	0.7722 (3)	0.6651 (2)	0.0731 (8)	
H10	0.4497	0.7186	0.6182	0.088*	
C11	0.2837 (4)	0.9231 (4)	0.6549 (2)	0.0777 (9)	
H11	0.3331	0.9734	0.6020	0.093*	
C12	0.1386 (4)	1.0006 (3)	0.7248 (3)	0.0782 (9)	
H12	0.0884	1.1037	0.7190	0.094*	
C13	0.0705 (4)	0.9233 (3)	0.8022 (2)	0.0686 (8)	
H13	−0.0272	0.9748	0.8491	0.082*	
C14	0.0666 (4)	0.6954 (3)	0.8999 (2)	0.0675 (7)	
H14A	0.1108	0.5915	0.8837	0.081*	
H14B	−0.0502	0.7374	0.9038	0.081*	
C15	0.0995 (4)	0.7078 (4)	1.0100 (2)	0.0785 (9)	
H15A	0.0642	0.8119	1.0222	0.094*	
H15B	0.0340	0.6697	1.0668	0.094*	
C16	0.2730 (4)	0.6270 (4)	1.0199 (3)	0.0827 (9)	
H16A	0.3399	0.6628	0.9625	0.099*	
H16B	0.3082	0.5220	1.0108	0.099*	
C17	0.2978 (4)	0.6479 (4)	1.1299 (3)	0.0867 (10)	
H17A	0.2674	0.7513	1.1377	0.130*	
H17B	0.4104	0.5924	1.1341	0.130*	
H17C	0.2311	0.6131	1.1868	0.130*	

### Atomic displacement parameters (Å^2^)

**Table:**

	*U*^11^	*U*^22^	*U*^33^	*U*^12^	*U*^13^	*U*^23^
Ni1	0.0448 (3)	0.0471 (3)	0.0603 (3)	−0.0124 (2)	−0.0012 (2)	−0.0050 (2)
S1	0.0525 (4)	0.0452 (4)	0.0670 (4)	−0.0135 (3)	0.0015 (3)	−0.0008 (3)
S2	0.0567 (4)	0.0489 (4)	0.0689 (4)	−0.0174 (3)	0.0060 (3)	−0.0021 (3)
S3	0.0525 (4)	0.0501 (4)	0.0736 (4)	−0.0146 (3)	0.0055 (3)	−0.0022 (3)
S4	0.0579 (4)	0.0488 (4)	0.0742 (5)	−0.0127 (3)	0.0096 (4)	−0.0021 (3)
N1	0.0769 (17)	0.0505 (15)	0.0934 (19)	−0.0155 (13)	−0.0048 (14)	−0.0097 (13)
N2	0.0728 (18)	0.0701 (17)	0.0859 (18)	−0.0194 (14)	0.0192 (15)	−0.0071 (14)
N3	0.0788 (18)	0.0535 (16)	0.103 (2)	−0.0110 (14)	0.0082 (15)	−0.0122 (14)
N4	0.0702 (18)	0.0761 (18)	0.0905 (19)	−0.0213 (14)	0.0174 (16)	−0.0071 (15)
N5	0.0505 (12)	0.0568 (13)	0.0561 (12)	−0.0137 (10)	−0.0055 (10)	−0.0116 (10)
C1	0.0588 (17)	0.0468 (14)	0.0640 (16)	−0.0144 (12)	−0.0012 (14)	−0.0049 (12)
C2	0.0487 (14)	0.0487 (14)	0.0529 (13)	−0.0139 (11)	−0.0031 (12)	−0.0079 (11)
C3	0.0610 (17)	0.0499 (17)	0.0608 (15)	−0.0200 (13)	−0.0031 (13)	−0.0034 (12)
C4	0.0512 (15)	0.0477 (14)	0.0581 (14)	−0.0143 (11)	−0.0040 (12)	−0.0104 (12)
C5	0.0555 (17)	0.0548 (16)	0.0709 (18)	−0.0164 (13)	0.0016 (15)	−0.0049 (13)
C6	0.0425 (14)	0.0557 (15)	0.0617 (15)	−0.0135 (11)	−0.0023 (12)	−0.0097 (12)
C7	0.0469 (14)	0.0512 (15)	0.0615 (15)	−0.0125 (12)	−0.0024 (12)	−0.0091 (12)
C8	0.0583 (17)	0.0542 (17)	0.0709 (17)	−0.0181 (14)	0.0033 (14)	−0.0048 (14)
C9	0.0559 (17)	0.0590 (16)	0.0660 (17)	−0.0105 (13)	−0.0073 (14)	−0.0122 (14)
C10	0.0630 (18)	0.075 (2)	0.0645 (17)	−0.0190 (16)	0.0043 (15)	−0.0130 (15)
C11	0.086 (2)	0.081 (2)	0.0653 (17)	−0.0384 (18)	−0.0079 (17)	0.0008 (16)
C12	0.090 (2)	0.0564 (17)	0.0769 (19)	−0.0210 (16)	−0.0151 (18)	−0.0030 (15)
C13	0.0653 (18)	0.0539 (16)	0.0689 (17)	−0.0092 (14)	−0.0057 (15)	−0.0130 (14)
C14	0.0621 (18)	0.0705 (18)	0.0689 (17)	−0.0297 (15)	−0.0060 (14)	−0.0053 (14)
C15	0.072 (2)	0.078 (2)	0.0735 (18)	−0.0302 (16)	0.0003 (16)	0.0052 (16)
C16	0.083 (2)	0.071 (2)	0.082 (2)	−0.0255 (17)	−0.0114 (18)	0.0043 (16)
C17	0.096 (2)	0.093 (2)	0.079 (2)	−0.046 (2)	−0.0262 (19)	0.0118 (18)

### Geometric parameters (Å, °)

**Table:**

Ni1—S1	2.1501 (8)	C9—C10	1.359 (4)
Ni1—S2	2.1436 (8)	C9—H9	0.9300
Ni1—S3	2.1458 (8)	C10—C11	1.361 (4)
Ni1—S4	2.1461 (8)	C10—H10	0.9300
S1—C2	1.715 (2)	C11—C12	1.384 (4)
S2—C4	1.721 (3)	C11—H11	0.9300
S3—C6	1.717 (3)	C12—C13	1.364 (4)
S4—C7	1.719 (3)	C12—H12	0.9300
N1—C3	1.145 (3)	C13—H13	0.9300
N2—C1	1.139 (4)	C14—C15	1.536 (4)
N3—C8	1.141 (4)	C14—H14A	0.9700
N4—C5	1.143 (3)	C14—H14B	0.9700
N5—C13	1.341 (4)	C15—C16	1.485 (4)
N5—C9	1.344 (3)	C15—H15A	0.9700
N5—C14	1.483 (3)	C15—H15B	0.9700
C1—C2	1.439 (4)	C16—C17	1.527 (5)
C2—C4	1.348 (4)	C16—H16A	0.9700
C3—C4	1.435 (4)	C16—H16B	0.9700
C5—C6	1.432 (4)	C17—H17A	0.9600
C6—C7	1.343 (4)	C17—H17B	0.9600
C7—C8	1.440 (4)	C17—H17C	0.9600
			
S2—Ni1—S3	86.97 (3)	C11—C10—H10	119.8
S2—Ni1—S4	179.28 (3)	C10—C11—C12	118.9 (3)
S3—Ni1—S4	92.59 (3)	C10—C11—H11	120.6
S2—Ni1—S1	92.34 (3)	C12—C11—H11	120.6
S3—Ni1—S1	177.33 (3)	C13—C12—C11	119.0 (3)
S4—Ni1—S1	88.08 (3)	C13—C12—H12	120.5
C2—S1—Ni1	103.03 (9)	C11—C12—H12	120.5
C4—S2—Ni1	103.36 (9)	N5—C13—C12	121.3 (3)
C6—S3—Ni1	102.87 (10)	N5—C13—H13	119.3
C7—S4—Ni1	102.92 (9)	C12—C13—H13	119.3
C13—N5—C9	119.7 (3)	N5—C14—C15	111.1 (2)
C13—N5—C14	119.5 (2)	N5—C14—H14A	109.4
C9—N5—C14	120.7 (2)	C15—C14—H14A	109.4
N2—C1—C2	178.1 (3)	N5—C14—H14B	109.4
C4—C2—C1	121.1 (2)	C15—C14—H14B	109.4
C4—C2—S1	121.02 (19)	H14A—C14—H14B	108.0
C1—C2—S1	117.88 (19)	C16—C15—C14	114.5 (3)
N1—C3—C4	178.3 (3)	C16—C15—H15A	108.6
C2—C4—C3	122.3 (2)	C14—C15—H15A	108.6
C2—C4—S2	120.21 (19)	C16—C15—H15B	108.6
C3—C4—S2	117.5 (2)	C14—C15—H15B	108.6
N4—C5—C6	179.3 (3)	H15A—C15—H15B	107.6
C7—C6—C5	121.6 (2)	C15—C16—C17	111.7 (3)
C7—C6—S3	120.9 (2)	C15—C16—H16A	109.3
C5—C6—S3	117.5 (2)	C17—C16—H16A	109.3
C6—C7—C8	120.0 (2)	C15—C16—H16B	109.3
C6—C7—S4	120.7 (2)	C17—C16—H16B	109.3
C8—C7—S4	119.3 (2)	H16A—C16—H16B	107.9
N3—C8—C7	176.4 (3)	C16—C17—H17A	109.5
N5—C9—C10	120.7 (3)	C16—C17—H17B	109.5
N5—C9—H9	119.6	H17A—C17—H17B	109.5
C10—C9—H9	119.6	C16—C17—H17C	109.5
C9—C10—C11	120.3 (3)	H17A—C17—H17C	109.5
C9—C10—H10	119.8	H17B—C17—H17C	109.5
